# Rare variant association study of veteran twin whole-genomes links severe depression with a nonsynonymous change in the neuronal gene *BHLHE22*

**DOI:** 10.1080/15622975.2021.1980316

**Published:** 2021-11-29

**Authors:** Daniel Hupalo, Christopher W. Forsberg, Jack Goldberg, William S. Kremen, Michael J. Lyons, Anthony R. Soltis, Coralie Viollet, Robert J. Ursano, Murray B. Stein, Carol E. Franz, Yan V. Sun, Viola Vaccarino, Nicholas L. Smith, Clifton L. Dalgard, Matthew D. Wilkerson, Harvey B. Pollard

**Affiliations:** a The American Genome Center, Collaborative Health Initiative Research Program, and Department of Anatomy, Physiology and Genetics, Uniformed Services University, Bethesda, MD, USA;; b Seattle Epidemiologic Research and Information Center, Office of Research and Development, U.S. Department of Veteran Affairs, Seattle, WA, USA;; c Department of Epidemiology, University of Washington, Seattle, WA, USA;; d Department of Psychiatry and of Family Medicine & Public Health, University of California, La Jolla, CA, USA;; e VA San Diego Center of Excellence for Stress and Mental Health, San Diego, CA, USA;; f Department of Psychological & Brain Sciences, Boston University, Boston, MA, USA;; g Department of Psychiatry, Uniformed Services University, Bethesda, MD, USA;; h Department of Epidemiology, Emory University, Atlanta, GA, USA;; i Department of Anatomy, Physiology and Genetics, Uniformed Services University, Bethesda, MD, USA

**Keywords:** MDD, GWAS, rare variant, BHLHe22, depression, whole-genome sequencing

## Abstract

**Objectives:**

Major Depressive Disorder (MDD) is a complex neuropsychiatric disease with known genetic associations, but without known links to rare variation in the human genome. Here we aim to identify rare genetic variants associated with MDD using deep whole-genome sequencing data in an independent population.

**Methods:**

We report the sequencing of 1,688 whole genomes in a large sample of male-male Veteran twins. Depression status was classified based on a structured diagnostic interview according to *DSM-III-R* diagnostic criteria. Searching only rare variants in genomic regions from recent GWAS on MDD, we used the optimised sequence kernel association test and Fisher’s Exact test to fine map loci associated with severe depression.

**Results:**

Our analysis identified one gene associated with severe depression, basic helix loop helix e22 (*P*_Adjusted_ = 0.03) via SKAT-O test between unrelated severely depressed cases compared to unrelated non-depressed controls. The same gene *BHLHE22* had a non-silent variant rs13279074 (*P*_Adjusted_ = 0.032) based on a single variant Fisher’s Exact test between unrelated severely depressed cases compared to unrelated non-depressed controls.

**Conclusion:**

The gene *BHLHE22* shows compelling genetic evidence of directly impacting the severe depression phenotype. Together these results advance understanding of the genetic contribution to major depressive disorder in a new cohort and link a rare variant to severe forms of the disorder.

## Introduction

Major Depressive Disorder (MDD) is a psychiatric condition caused, in part, by a complex network of heritable risk factors that scale with severity (Sullivan et al. 2000; Levinson 2006). MDD is estimated to affect one in six adults in their lifetime (Otte et al. 2016). Recent genome-wide-association studies (GWAS) have identified genomic regions that may contain genetic variants that could functionally contribute to the heritability of this disorder (Hyde et al. 2016; Wray et al. 2018; Howard et al. 2019) These GWAS efforts rely on utilising array-based technology which samples only common variation, which are unlikely to have functional links to disease and fail to explain the full heritability of MDD (Auer and Lettre 2015). Similarly, commonly studied depression candidate genes have yet to be conclusively linked to functional changes that directly cause increased risk of MDD (Border et al. 2019). Expanding to a whole-genome-sequence association study provides an opportunity to identify rare causative variants, which are overlooked by common-variant array-based genetic association studies.

The Vietnam Era Twin (VET) Registry is a cohort of male-male twin pairs maintained since the 1980s, many of whom served during the Vietnam era, and includes physiologic and psychological measures as well as biospecimens from its members (Eisen et al. 1987). In 2001 a special biorepository was created, comprising whole-blood samples collected from a subset of 1819 twins, mostly paired siblings. It was this special biorepository of twin-pairs that became the source of DNA samples used for the WGS study reported here. Previous VET Registry research has focussed on the heritability of MDD and a significant genetic influence was found for the most severe types of depression (Lyons et al. 1998). In that study, depression phenotype variables were collected using the *Diagnostic Interview Schedule III Revised (DIS-III-R)* which assesses *Diagnostic and Statistical Manual-III-R (DSM-III-R)* diagnostic criteria via telephone interview. This same data and samples are used for the current study and defined the scope of cases and controls for each phenotype.

One of the primary challenges of a genetic association study is achieving adequate statistical power necessary to identify associations with small effect sizes. Many current WGS cohorts, including the one presented here, lack sufficiently large sample sizes needed to identify low frequency genetic associations with genome-wide significance. However, many array-based studies which have identified regions associated with MDD using millions of individuals have narrowed the search space for potential casual variants. We hypothesised that rare, potentially causal variation for severe MDD could be discovered by analysing the genomic regions previously identified by GWAS. In contrast to correcting for multiple testing by using thresholds for genome-wide studies, controlling for the total number of tests performed by limiting to these regions (‘testing wide significance’) provides appropriate control for this study design and provides power for this small cohort. As hypothesised, this type of focussed approach successfully linked severe MDD to a non-silent variant on chromosome 8, in the single exon of the basic helix loop helix e22 (*BHLHE22*) gene.

## Methods

### Sample collection

The cohort for this investigation was drawn from the Vietnam-Era Twins (VET) Registry. The VET Registry contains 7369 male-male twin pairs (14,738 individuals) in which one or both brothers served in the United States military during the Vietnam Era (1965–1975). Informed consent was obtained from all individual participants involved in the study, and all experiments were performed in accordance with relevant guidelines and regulations. Full details about the construction of the VET Registry have been previously published (Eisen et al. 1987). The VET Registry biorepository is a subsample of 1,819 members whose blood was collected during previous in-person studies. The entire biospecimen repository was sequenced and samples in this cohort were sequenced without regard to depression status, twin status, or batching.

There are 4 depression phenotypes variables in the VET registry. These depression variables were derived from the *Diagnostic Interview Schedule-III-R (DIS-III-R)* depression module according to the *Diagnostic and Statistical Manual—III-Revised (DSM-III-R)* criteria. This structured telephone interview was administered between 1991 and 1994 as part of the Harvard Drug Study (Tsuang et al. 2001). Participants were not selected on the basis of any substance use or other diagnostic characteristic. Responses and diagnostic criteria from the *DIS-III-R* were used to define four different values that reflect the lifetime history of depression for each individual within the cohort up to 1994. The first variable is the lifetime history of MDD diagnosis. The second variable is the count of depression symptoms experienced during a member’s lifetime, ranging from 0 to 9 of 9 symptoms experienced. The third variable records the age at which the individual first experienced a depressive episode, which then informs a binary classification denoting early-onset (age < 30) or late onset (age > 30). The last variable is the *DSM-III-R* classification of the severity of the depressive episode experienced among those with a lifetime diagnosis of depression. This 3-level classification within the *DIS-III-R* is as follows: *mild*- meaning few, if any symptoms more than the minimum to make the diagnosis and only minor impairment in usual social activities or relationships; *moderate*-meaning impairment and symptoms between mild and severe; and *severe/severe-psychotic*-meaning several symptoms more than the minimum required for the diagnosis, marked by interference with occupational or social functioning with the potential for additional psychosis-related delusions or hallucinations. All individuals labelled as severely depressed within the VET Registry and the VET Registry Biorepository were without additional psychosis symptoms.

### Sequencing and genotyping

Samples of DNA were housed at the Department of Veteran Affairs (VA) Massachusetts Veterans Epidemiology Research and Information Centre (MAVERIC) biorepository and were sent to The American Genome Centre at Uniformed Services University. They were then sequenced on an Illumina HiSeq X System using paired-end 151 bp read lengths targeting 400 million total reads. Sequenced reads were processed by the Illumina HiSeq Analysis Software version 2.0 (2.5.55.13). Variants across all samples in the cohort were merged and normalised. Population level autosomal variant sites were then filtered using two conditions: (1) the proportion of samples with non-reference alleles having a PASS filter is at least 90%; and (2) the proportion of samples having a minimum genotype quality (greater or equal to 20) of at least 90%. Variants within the cohort VCF were annotated using ANNOVAR (Wang et al. 2010). Reference population allele frequencies were obtained from TwinsUK, 1000 Genomes Project, GnomAD, and ExAC (1000 Genomes Project Consortium et al. 2015; Moayyeri et al. 2013; Lek et al. 2016). Non-silent mutations were assessed using data from SIFT, Polyphen2, MutationAssessor, PROVEAN, and fathmm (Adzhubei et al. 2010; Choi et al. 2012; Shihab et al. 2013; Vaser et al. 2016). Twin mate identities were verified by calculating identity by state (IBS) via rvtests (Zhan et al. 2016). To compare two samples’ genome-wide genotypes including non-reference and reference-matching genotypes, genome variant call format (GVCF) files were merged and analysed for concordance.

### Target regions for focussed analysis

We focussed on the most recent study of MDD with the largest sample size for the selection of our genome regions of interest (Howard et al. 2019). Using an initial list of 102 lead variants (substitutions and indels), we measured the size of the linkage disequilibrium (LD) block centred on each lead variant within the 1000 Genomes background populations of Americans of African Ancestry in SW USA (ASW) and Utah Residents (CEPH) with Northern and Western European Ancestry (CEU) to estimate a similar composition of ancestry compared to the known ancestry of our cohort using the LDLink tool (Machiela and Chanock 2015). Borders for each unique LD block were set as the breadth of the peak which was greater than 0.2 *r*^*2*^ for each locus of interest. This method follows a similar example in the literature of a haplotype-based search in psychiatric disorders (Budde et al. 2019; Howard et al. 2019). The borders of the LD region for each associated locus from the set of 102 sites are listed in Supplemental Table 1.

### VET registry WGS cohort construction

This biorepository cohort and its resultant WGS data were conceived to serve a broad set of research goals, including but not limited to associations with MDD. It was therefore necessary to sequence the full biorepository which included monozygotic and dizygotic twins. To account for the presence of related data we therefore intended to balance the need to include the maximum amount of unique DNA and cases, with the need to exclude samples for familial relatedness. Within the literature there are multiple strategies for dealing with genomic data from related pairs in genetic association studies. These include genome-wide association study designs such as including all genomic data from all twin pairs (Valdes et al. 2008; Verweij et al. 2010; Miller et al. 2012), including both DZ twins and removing one MZ twin (Wallace et al. 2008; Loukola et al. 2014), and not including any genomic information from the paired twin when it was available (Luciano et al. 2011; Kettunen et al. 2012; Armour et al. 2014). The final cohort as defined by striking one individual from each MZ pair, including both individuals of a DZ pair, and including all singletons. In addition, the cohort was limited to European individuals. This resulted in a cohort of up to *N* = 1060 individuals. Samples with missing phenotype data were excluded. To account for the addition of relatedness by the inclusion of DZ pairs in the cohort, we constructed a more conservative follow up cohort which included only unrelated individuals of European ancestry, and controls with no history of depression (*n* = 676). Peddy was used to measure the coefficient of relatedness between unrelated individuals. No individuals with a coefficient of relatedness ≥0.1 were present in the unrelated cohort, as expected for a coefficient of relatedness between unrelated pair comparisons (Manichaikul et al. 2010; Pedersen and Quinlan 2017).

### Genetic association testing

Association testing was conducted across each gene and non-silent variant within the 102 previously associated autosomal loci. Association at each gene was measured using the optimal sequence kernel association test, SKAT-O (Lee et al. 2012). Single-variant enrichment was evaluated using Fisher’s Exact Test. Statistical significance was determined after correction for multiple testing by using a Bonferroni adjusted *p* value based on the number of genes tested. Each of the four *DSM-III-R* MDD diagnostics variables was measured for enrichment in genes and single variants independently of one another, similar to previous VET Registry research (Lyons et al. 1998). Similar approaches in non-depression research have shown precedent for testing related but independent phenotypes across an exome (Haddad et al. 2016).

Prior to testing, variants were filtered to those with a MAF < 0.05, those that are non-silent, and those that have an allele count of at least three alternative alleles. ‘Non-silent’ was defined as the following classes of variant as annotated by ANNOVAR: nonsynonymous, frameshift, stopgain, and stoploss. Principal components 1–4 were used as covariates to account for population structure for the SKAT-O. Four MDD categories were tested for association. These variables included lifetime diagnosis of a major depressive episode, severity of depression, the number of symptoms experienced, and early onset of MDD. Plotting of variant association was performed using LDAssoc (Machiela and Chanock 2018). Track data for brain tissue expression was sourced from Genotype-Tissue Expression (GTEx) project (GTEx Consortium 2013).

## Results

### Sequencing the VET registry WGS cohort allows the inclusion of genomic data from related pairs

We sequenced blood-derived DNA from 1688 male VET Registry members with a resulting mean read depth of 43.7x. These high-quality whole-genome sequences were sourced from biorepository samples of 982 monozygotic (MZ) twins, 632 dizygotic (DZ) twins, and 74 unrelated individuals in the VET Registry, whose blood was collected between the years of 2002 and 2013 (Table 1, Supplementary Figure 1). Three duplicate pairs of DNA samples were also sequenced as technical replicates. Phenotype data described self-reported ancestry and four MDD *DSM-III-R* variables.

To better understand the population structure of the genomes sourced from the VET Registry, we conducted a principal component (PC) analysis including the first 20 eigenvectors computed from cohort-wide variation data (Supplemental Figure 2). The plots of eigenvectors for the VET Registry cohort closely resemble genome-wide PC distributions from previous studies of global human populations (1000 Genomes Project Consortium et al. 2015), and revealed no biases due to reagent batch, collection date, or other potential confounding effects.

### Twin genome variation reflects zygosity status

**Figure 1.**
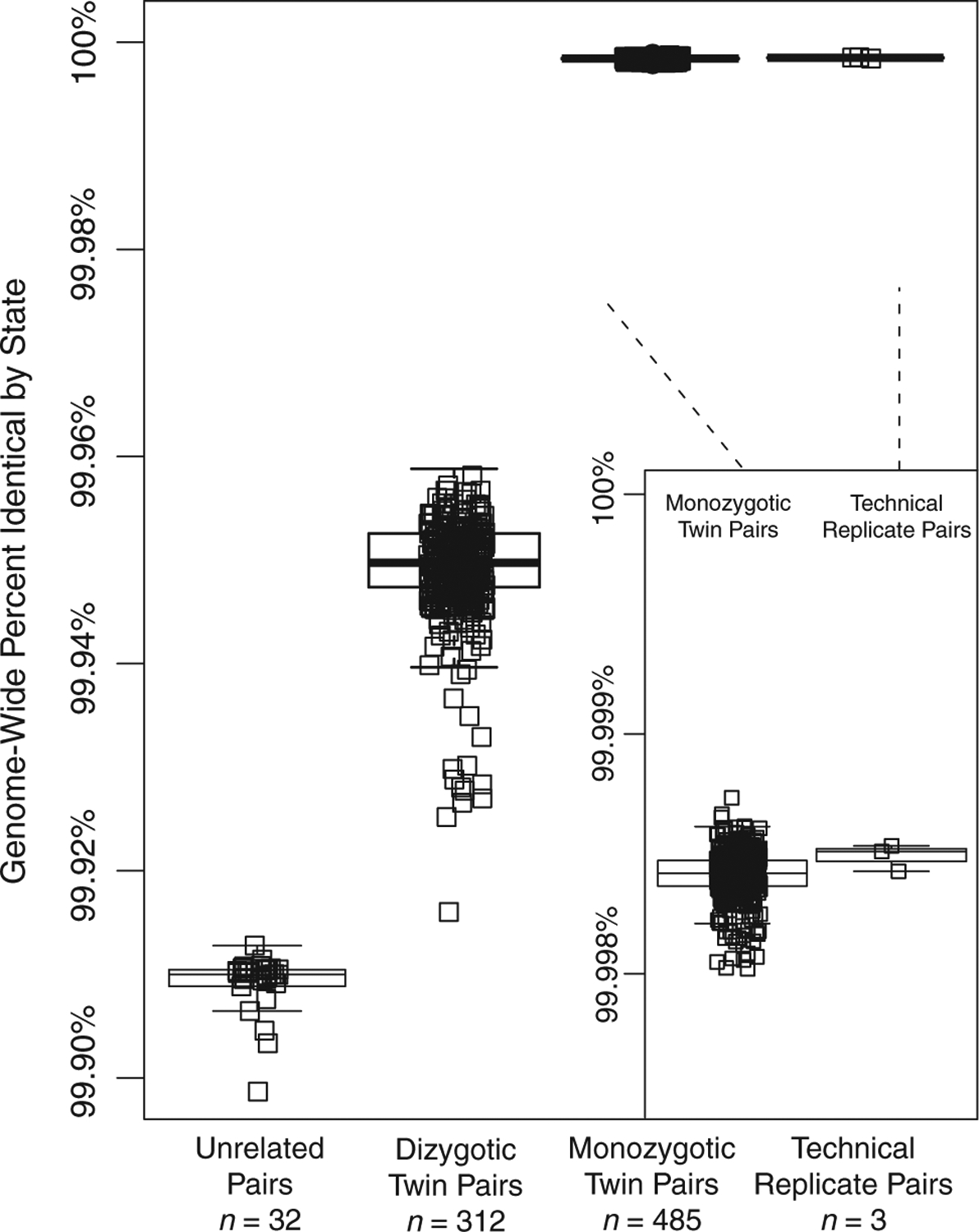
Genome-wide identity by state (IBS) between twin pairs for a cohort of monozygotic, dizygotic, unrelated individuals, and technical replicate whole-genome sequences. Percent IBS for each twin pair, technical replicates, and randomly paired unrelated individuals is listed as a box plot. The centre and ends of the boxes represent the 25th, 50th and 75th percentiles of IBS values and the whiskers on each plot represent the range of IBS within 1.5 times the interquartile range of the lower and upper quartiles. Each individual IBS value is also plotted. Inset is a plot of genome-wide percent identity by state for monozygotic twins and technical replicates, respectively.

Having whole-genome sequence data of monozygotic twins created the opportunity to investigate the nature of twin genetics. Recent studies have suggested that monozygotic twins may harbour rare variation that is somatic in nature, and that sesquizygotic twinning can occur, albeit rarely (Nishioka et al. 2018; Gabbett et al. 2019). Given the large number of MZ twins within the VET Registry cohort, we explored the genome-wide concordance of alleles between all pairs of monozygotic, dizygotic, replicate and unrelated samples within the cohort (Figure 1). On average we detected 3,094,980,951 co-genotyped sites between two individuals. Monozygotic twins showed nearly the same percentage of identical alleles (mean 99.99841%, *n* = 485 pairs) as technical replicates (mean 99.99849%, *n* = 3). Dizygotic twins also displayed high allelic similarity (mean 99.948%, *n* = 312 pairs) although that similarity was reduced compared with monozygotic twins. Both monozygotic and dizygotic twins had significantly greater similarity compared with paired unrelated individuals (mean 99.91%, *n* = 32 randomly assigned pairs). Among the population of monozygotic twins, we identified 596 homozygous non-silent variants discordant between pairs of MZ twins (*n* = 491 pairs); this included non-synonymous variants, frameshifts, stop-loss, and stop-gains. Of this set, 132 unique non-silent variants had a functional impact (CADD) score, of at least 20. Gene ontology analysis of these 132 disruptive sites between MZ twins did not reveal any remarkable gene sets. In summary, the cohort of twins matched expectations of relatedness and does not show any clusters of non-silent mutations.

### Gene-collapsed rare variant analysis identifies non-silent burden associated with MDD

To determine if genes in the 102 regions previously associated with MDD might have an enrichment of non-silent rare variants, we utilised gene-collapsed SKAT-O testing (Lee et al. 2012). A total of 340 genes exist within the borders of the 102 regions, making them available for inclusion in a gene-wise SKAT-O analysis. The analysis was conducted using one test per gene for each of the four phenotype variables (lifetime depression diagnosis, early age-of-onset, symptom count, severe depression). Of these 102 regions known to be associated, 22 regions had no genes present. For the severe depression phenotype, the resulting testing space of 80 regions contained a total of 212 genes with at least 3 non-silent variants, upon which the tests were conducted. This test included *n* = 47 severely depressed individuals compared to *n* = 1007 non-severely-depressed European individuals. Full results are reported in Supplemental Table 2. We found that the developmental transcription factor *BHLHE22* was significantly enriched for rare variation among individuals with severe depression (SKATO *P* = 0.000123, *P*_Adjusted_ = 0.027, Table 2, Supplemental Table 2A). This basic helix loop helix transcription factor contained 21 non-silent variants across the gene on chromosome 8. No other significant associations were found (Supplemental Table 2C–H). By limiting the cohort to severely depressed unrelated individuals compared to unrelated individuals with no history of depression (*n* = 676), we found that *BHLHE22* continued to have a significant enrichment of rare, non-silent variants (SKATO *P* = 0.000149, *P*_Adjusted_ = 0.032) (Supplemental Table 3A).

### Single variant analysis identifies a non-silent rare variant associated with MDD

We also performed single variant analysis on all non-silent, rare (MAF ≤ 0.05) variants within the borders of the 102 regions for four different MDD-related variables (lifetime depression diagnosis, age-of-MDD onset before 30, symptom count 0–9, and depression severity). In the case of severe depression, 548 tests were performed, one for each non-silent variant. As utilised above, this test included *n* = 47 severely depressed individuals compared to *n* = 1007 non-severely-depressed European individuals. One non-silent variant, (rs13279074) *BHLHE22*, was significantly associated with severe depression (Fisher’s Exact *P* = 6.98 × 10^−05^, *P*_Adjusted_ = 0.038, Table 2, Supplemental Table 2B). The allele frequency for rs13279074 among severe MDD cases was 0.08 compared to 0.009 for those without severe depression, odds ratio: 8.45 (95% CI 3.4614–20.6404), an odds ratio similar in size to other reported rare-variant associations (Fritsche et al. 2016; Pigors et al. 2018). This variant at rs13279074 encodes at G>T change in the first and only exon of the *BHLHE22* gene, also known as *BHLHB5*. This translates to a glycine to valine change at position 106 of the protein. To rule out relatedness or other cryptic effects, we reduced the cohort to unrelated individuals of European descent, by selecting one twin from each pair of MZ and DZ samples with priority for inclusion given to individuals diagnosed with MDD, and all singleton samples. We found that rs13279074 remained enriched when comparing severely depressed unrelated individuals to unrelated individuals with no history of depression (*n* = 676) (Fisher’s Exact *P* = 8.01 × 10^−05^, *P*_Adjusted_ = 0.032, Supplemental Table 3B). The rare variant in *BHLHE22* was statistically significant after Bonferroni correction in the large (*n* = 1,054) and small cohorts (*n* = 696).

### Regional context of rs13279074 in BHLHE22

**Figure 2.**
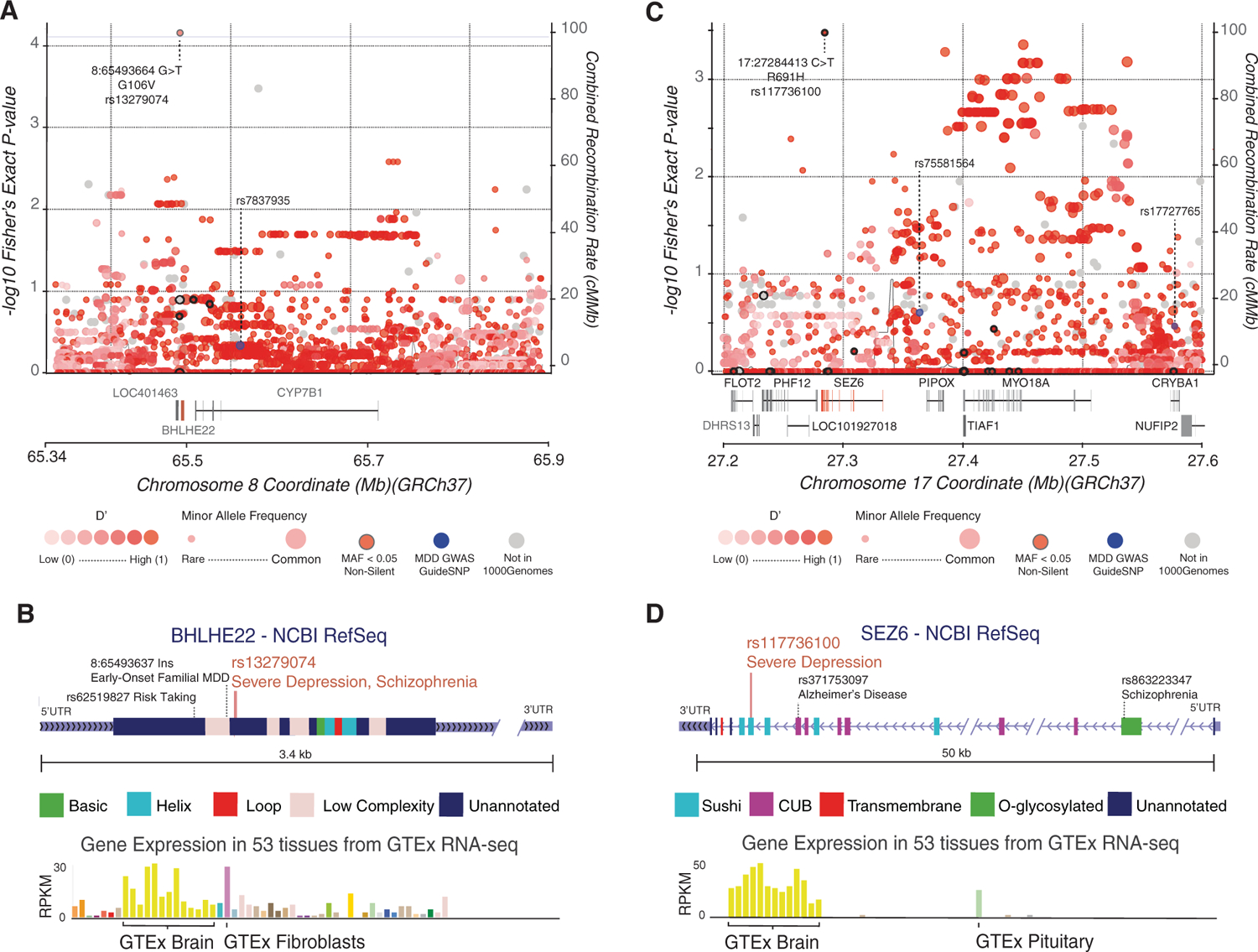
Regional displays of the BHLHE22 and SEZ6 loci and the single-variant associations at rs13279074 and rs117736100. (A) Variant associations to severe depression within the local region surrounding the BHLHE22 gene for rare non-silent variants (black border) as well common variants (no border). Shading of each circle is proportional to linkage disequilibrium, D′ from the 1000 Genomes cohort. The size of the circle is proportional to Minor Allele Frequency. Recombination rate and overlapping gene models are also shown. Labeled loci are previously described common-variant associations to MDD and the variant of interest. (B) Gene structure display of BHLHE22 with protein domains and region annotation labelled. Two other previously-reported associated variants with phenotypes related to MDD are displayed. Expression data describing the tissue-specific expression profile of BHLHE22 is sourced from the Genotype-Tissue Expression project, most prevalent tissues are labelled. (C) Plot of variant associations to severe depression within the local region surrounding the SEZ6 gene. Labels and descriptions correspond to those seen in Figure 2A. (D) Gene structure display of SEZ6 with protein domains and region annotation labelled where available.

To better understand the context of the association of rs13279074 with severe depression, we plotted variant associations for all rare and common variants across its parent genome region on chromosome 8 (Figure 2A). Among rare, non-silent variants in this region, only rs13279074 had significance. Linkage disequilibrium sourced from the 1,000 Genomes cohort showed an extended block of linkage and an elevated group of p-values centred on the previously reported lead MDD SNP – rs7837935. The novel rare variant present in *BHLHE22* appears to be within the same block of inheritance.

Further context analysis of rs13279074 within the *BHLHE22* gene supports a functional role in MDD (Figure 2B). Among the 35 human tissues assayed by the Genotype-Tissue Expression project (GTEx), *BHLHE22* was found to be primarily expressed in brain tissue and research has shown it is involved in the development of the neocortex (Joshi et al. 2008; GTEx Consortium 2013). The gene has one exon with three annotated protein domains corresponding to the common basic helix loop helix protein structure (Xu et al. 2002). The associated rare variant rs13279074 is located outside the annotated BHLH domains. However, the associated change of a glycine to a valine found at position 106 is conserved across species and does not overlap with the known low complexity regions of the protein. Additionally, *BHLHE22* forms a repressor complex with the *PRDM8* protein, whose protein-protein interaction site is not yet understood and may overlap with this association with severe depression (Ross et al. 2012). Multiple studies have linked other variants in *BHLHE22* with neurological phenotypes and disease, including increased risk taking, and early-onset familial MDD (Subaran et al. 2016; Clifton et al. 2018). Lastly, we find that rs13279074, through imputation, is associated with Schizophrenia (*P* = 0.0004 *n* = 96,499) within the Finnish genetics cohort (FinnGen Freeze 2) database (mFinnGen 2020), providing additional psychiatric disease-association evidence for this rare variant.

Across the VET Registry cohort, the variant (rs13279074) *BHLHE22* was observed 41 times across 3376 total alleles, 37 of which were in a heterozygous context, and 4 alleles which were in two homozygous individuals. The two homozygous individuals are related monozygotic twins, one of whom was diagnosed with severe depression and the other was not clinically diagnosed with severe MDD but exhibited a high depression symptom count (8/9 symptoms and 7/9 symptoms respectively). In this cohort the *BHLHE22* gene contained 20 other non-silent variants, four of which met the threshold for testing (minor allele count >3, minor allele frequency <0.05). Among that set of four, the severe depression associated variant rs13279074 had the highest CADD phred score, measuring 23.5. Classification of the amino acid change at position 106 was mixed, with Polyphen2 and fathmm predicting a deleterious classification and SIFT, MutationAssessor and PROVEAN predicting a tolerated effect. As previously stated, among cases of severe depression, the allele frequency for rs13279074 was found to be 0.08 compared to 0.009 for individuals without severe depression. Among all samples in the VET Registry cohort, the allele frequency was 0.012 for rs13279074. This is comparable to the allele frequency seen in large, unselected populations, which range between 0.002 and 0.02 with a mean of 0.01169 (Karczewski et al. 2020). Together, this evidence shows that the rare variant rs13279074 is significantly associated with severe depression, and imparts a protein change at a conserved site in a neurological gene that has been classified as disruptive.

### Non-silent variant rs117736100 in SEZ6

The second most enriched single variant was the non-silent rare variant rs117736100, which was enriched for severe depression but not significant after correction (Fisher’s Exact *P* = 0.0003, *P*_Adjusted_ = 0.18, Table 2, Supplemental Table 2B). The MAF for rs117736100 among severe MDD cases was 0.027 compared to 0.0004 in those without severe MDD, odds ratio: 66.3626 (95% CI 6.8359–644.2420). This variant at rs117736100 encodes a C > T nucleotide change in the gene, Seizure-Related-6 (*SEZ6*; rs117736100). The number of observations of rs117736100 in *SEZ6* were too few to permit testing in an unrelated cohort. In the case of the novel rare variant observed in *SEZ6*, rs117736100 occurs in a region of elevated significance surrounding a block of inheritance previously associated with MDD (Figure 2C). As seen in Figure 2D, the rare variant rs117736100 in *SEZ6* is expressed in the brain and present in a SUSHI/SCR domain.

## Discussion

The primary finding in this paper links severe depression, a phenotype previously found to carry the high heritability of disease, to a rare non-silent change in the neuronal transcription factor *BHLHE22*. This non-silent variation at position 106 is predicted to cause a nonsynonymous change in a region of the protein predicted to be conserved and outside of known low complexity regions. The *BHLHE22* rare variant rs13279074 has additional properties consistent with a possible effect on neuropsychiatric disorders. In brief, the rare variant is predicted to be disruptive: it occurs in a gene that is specific to neurons in the developing neocortex; it occurs at a higher rate than expected in severely depressed individuals; it occurs in a gene linked to multiple psychological diseases including familial MDD; and in an external cohort, the same variant rs13279074 was found to also be enriched among individuals diagnosed with Schizophrenia. Most importantly, this variant overlaps the same linkage block on chromosome 8 as those seen in the most recent MDD association studies. In addition to this site, we also found a promising SNP, rs117736100, in the *SEZ6* gene, which shares many of the same lines of evidence as the *BHLHE22* association but fell short of significance. Together these novel rare variant associations show that refinement of the current understanding of MDD genetics is possible and is guided by the previous literature on inheritance and association.

*BHLHE22* is primarily expressed in the brain, yet its mechanism of influence on MDD is not fully understood. However, multiple lines of evidence relate the *BHLHE22* gene to psychiatric disease. These include being a risk factor for schizophrenia (Hennig et al. 2017), PTSD (Gelernter et al. 2019), familial depression (Subaran et al. 2016) and risk-taking-behavior (Karlsson Linner et al. 2019). In particular, the imputed association of rs13279074 with schizophrenia within the Finngen2 database demonstrates the impact that changes in this gene and base pair can have on psychiatric disease. Functionally, *BHLHE22* drives neocortical arealization, the process by which individual areas of the neocortex develop their own cytoarchitecture, connectivity and function (Alfano and Studer 2013). *BHLHE22* also differs from other members of the class of Basic Helix-Loop-Helix genes in that it cannot bind directly to DNA and must have a binding partner that allows the complex to bind target regions. One such binding partner is the BHLH gene *TCF4*, and its paralog *TCF3*. The *BHLHE22*/*TCF4* complex has been shown to drive oligodendrocyte differentiation and CNS myelinisation (Wedel et al. 2020). *TCF4* has also recently been shown to be a schizophrenia risk gene on its own (Hennig et al. 2017). Thus the enhanced risk for schizophrenia associated with *BHLHE22* could be related to the influence or interaction with *TCF4*, and its downstream targets. *BHLHE22* also forms a transcriptional repressor complex with *PRDM8*, a member of a conserved family of methyltransferases that affects neuronal circuit assembly but whose influence on disease is unknown (Ross et al. 2012). This evidence suggests the mechanism of *BHLHE22* in causing psychiatric disease is through transcriptional regulation via partner genes which alters neuronal architecture. The downstream effects of these transcriptional regulators are many, and provide a plausible mechanism by which *BHLHE22* may contribute to a causal genetic determinate for severe MDD.

While not significantly enriched in this study, the variant rs117736100 in *SEZ6* may be a promising candidate for future study as it has several intriguing features. Previous characterisation of this gene in mice has shown that *SEZ6* acts to increase dendrite arborisation and excitability of neurons within the cortex (Gunnersen et al. 2007). There are multiple reported common variants in *SEZ6* which have been linked to diseases such as febrile seizure, epilepsy, Alzheimer’s disease and schizophrenia (Yu et al. 2007; Jiang et al. 2012; Ambalavanan et al. 2016; Paracchini et al. 2018). Additional studies have also linked homologs of *SEZ6*, such as *SEZ6L*, to bipolar disorder (Xu et al. 2013).

We acknowledge some limitations to this work. Association studies comprised of whole-genome sequences are an emerging field. As a result, sample sizes are small while cohorts are sequenced and slowly aggregated. This includes this study, which is a first of its kind in sequencing a large cohort of WGS data from individuals with psychiatric disease. While the size of the cohort falls short of a study in a mature field, we feel that this initial result will point the way towards further investigation of the genes and variants identified when larger cohorts are available in the coming years. This WGS approach specifically uses regions with known associations to MDD from deeply sampled array-based studies. It is a reasonable assumption that those regions are enriched for variants associated with major depressive disorder. Our reported genes and variants meet significance thresholds for the number of tests performed, which is appropriate given our hypotheses for investigating these regions; however, this threshold is less than genome or exome-wide significance. Individuals diagnosed with severe depression have been shown to have the strongest heritability of MDD (Lyons et al. 1998). However, the most severe forms of depression may also have a diverse and unique biology that differentiates it from the MDD observed in this cohort. Additionally, the adult all-male cohort may limit whether these observations apply to a general population, as it is known that MDD has higher prevalence among women and lower prevalence among the elderly (Piccinelli and Wilkinson 2000; Kessler et al. 2010). Lastly, due to the inherent rarity of the variation being studied, we acknowledge that for the *SEZ6* rs117736100 locus we report very low counts of severe depression cases and alternative alleles observed, resulting in the variant falling short of significance after correction for multiple testing. Despite these potential sources of bias, we feel that this investigation is a useful and meaningful step towards identifying the underlying genetics of MDD.

In conclusion, we have identified a rare variant in the *BHLHE22* gene linked to the most severe forms of MDD. This rare disruptive change forms a functional link between phenotype and genotype. Also, the evidence that cases of severe depression are an important resource for finding genetic MDD associations gives insight for the future search for additional novel links to this disorder.

## Supplementary Material

Supplement 1

Supplement 2

Supplement 3

Supplement 4

## Figures and Tables

**Table 1.** Clinical data and sequencing status from a repository cohort of 1,688 individuals sequenced from the Vietnam Era Twins Registry Biorepository.

	Data category	All	Monozygotic twins	Dizygotic twins	Non-reflexive twins/Singletons
Subject samples (*N* = 1747)	Kept WGS	1688	982	632	74
Ancestry (*N* = 1688)	EUR	1556	898	593	65
	AFR	110	70	34	6
	AMR	15	8	5	2
	EAS	7	6	0	1
Lifetime depression diagnosis (*N* = 1688)	No depression	1461	843	551	67
	Bereavement	14	8	6	0
*DSM-III-R* Depression diagnosis	Depression Dxn	182	107	71	4
	Missing data	31	24	4	3
Depression symptom count (0–9) (*N* = 1688)	0–8	1617	932	616	69
	9	39	25	12	2
*DSM-III-R* symptom count	No data	32	25	4	3
Depression severity (Mild-Severe) (*N* = 1688)	Not depressed	1529	893	565	71
	Mild	46	28	18	0
*DSM-III-R* severity	Moderate	55	27	26	2
	Severe	58	34	23	1
Depression age of onset (*N* = 1688)	No data	1508	877	561	70
	≤30	81	48	31	2
*DSM-III-R* age of onset	>30	99	57	40	2

In the case of severity and age of onset, no data was collected for individuals who were not diagnosed with depression. For the categories of Symptom Count, Severity, and Age of Onset the group ‘No Data’ refers to samples which had no value recorded for that category.

**Table 2.** Gene burden test and single variant results for the 102 previously associated regions for four depression phenotype variables.

A	Cases phenotype	Controls phenotype	Population	Sample inclusion	Tests performed	Cases/controls	MARKER_ID	Gene	Number of variants	*P* value	Bonferroni Adjusted *P* value
	**Severe depression**	**No Severe depression**	**EUR**	**One** MZ **twin excluded**	**226**	**47/1007**	**8:65493403–65494463**	***BHLHE22***	**28**	**0.000123**	**0.027798**
	Severe depression	No Severe depression	EUR	One MZ twin excluded	226	47/1007	3:44775962–44776626	*ZNF501*	8	0.016675	1
	Severe depression	No Severe depression	EUR	One MZ twin excluded	226	47/1007	6:27805753–27806078	*HIST1H2AK*	8	0.021831	1
	**Severe depression**	**No depression**	**EUR**	**No related samples**	**215**	**45/631**	**8:65493403–65494463**	***BHLHE22***	**28**	**0.000149**	**0.03207155**
	Severe depression	No depression	EUR	No related samples	215	45/631	11:65561574–65562686	*OVOL1*	4	0.033921	1
	Severe depression	No depression	EUR	No related samples	215	45/631	8:65509212–65711089	*CYP7B1*	14	0.041691	1
	Depression DXN	No depression	EUR	One MZ twin excluded	226	150/909	6:100957344–101296389	*ASCC3*	25	0.001597	0.360809
	Depression DXN	No depression	EUR	One MZ twin excluded	226	150/909	13:99109476–99174089	*STK24*	5	0.007886	1
	Depression DXN	No depression	EUR	One MZ twin excluded	226	150/909	6:27805753–27806078	*HIST1H2AK*	8	0.019386	1
	Early MDD onset	No early onset	EUR	One MZ twin excluded	227	65/995	3:49249233–49294503	*CCDC36*	9	0.004627	0.6436812
	Early MDD onset	No early onset	EUR	One MZ twin excluded	227	65/995	3:49548008–49570583	*DAG1*	20	0.027113	1
	Early MDD onset	No early onset	EUR	One MZ twin excluded	227	65/995	6:100957344–101296389	*ASCC3*	25	0.028526	1
	At least 9 symptoms	Less than 9 symptoms	EUR	One MZ twin excluded	226	32/1025	18:52585211–52609922	*CCDC68*	11	0.002052	0.4637972
	At least 9 symptoms	Less than 9 symptoms	EUR	One MZ twin excluded	226	32/1025	6:27805753–27806078	*HIST1H2AK*	8	0.008057	1
	At least 9 symptoms	Less than 9 symptoms	EUR	One MZ twin excluded	226	32/1025	6:100957344–101296389	*ASCC3*	25	0.009764	1
B	Cases phenotype	Controls phenotype	Population	Sample inclusion	Tests performed	Cases/controls	Coordinates (Hg19)	Gene	dbSNP Loci	*P* value	Bonferroni Adjusted *P* value
	**Severe depression**	**No severe depression**	**EUR**	**One MZ twin excluded**	**548**	**47/1007**	**8:65493664**	***BHLHe22***	**rs13279074**	**6.98E-05**	**0.0381806**
	Severe depression	No severe depression	EUR	One MZ twin excluded	548	47/1007	17:27284413	*SEZ6*	rs117736100	0.000333	0.1820279
	Severe depression	No severe depression	EUR	One MZ twin excluded	548	47/1007	3:49694230	*BSN*	rs148674638	0.011133	1
	**Severe depression**	**No depression**	**EUR**	**No related samples**	**402**	**45/631**	**8:65493664**	***BHLHe22***	**rs13279074**	**8.01E-05**	**0.0322002**
	Severe depression	No depression	EUR	No related samples	402	45/631	20:40079594	*CHD6*	rs140007009	0.012584	1
	Severe depression	No depression	EUR	No related samples	402	45/631	14:103969253	*MARK3*	rs188709144	0.012584	1
	Depression DXN	No depression	EUR	One MZ twin excluded	547	150/909	8:65493664	*BHLHe22*	rs13279074	0.005165	1
	Depression DXN	No depression	EUR	One MZ twin excluded	547	150/909	17:27284413	*SEZ6*	rs117736100	0.010083	1
	Depression DXN	No depression	EUR	One MZ twin excluded	547	150/909	14:103969253	*MARK3*	rs188709144	0.022572	1
	Early MDD onset	No early onset	EUR	One MZ twin excluded	548	65/995	3:49293819	*CCDC36*	rs147025639	0.003939	1
	Early MDD onset	No early onset	EUR	One MZ twin excluded	548	65/995	2:86769377	*CHMP3*	rs144255258	0.006573	1
	Early MDD onset	No early onset	EUR	One MZ twin excluded	548	65/995	3:49308804	*C3orf62*	rs150162031	0.008349	1
	At least 9 symptoms	Less than 9 symptoms	EUR	One MZ twin excluded	548	32/1025	18:52605279	*CCDC68*	rs72928889	0.002909	1
	At least 9 Symptoms	Less than 9 symptoms	EUR	One MZ twin excluded	548	32/1025	3:49694230	*BSN*	rs148674638	0.005206	1
	At least 9 symptoms	Less than 9 symptoms	EUR	One MZ twin excluded	548	32/1025	3:48732733	*IP6K2*	rs200143726	0.008508	1

(A) Top three gene results of the optimised sequence kernel association test (SKAT-O) are displayed for four different phenotype comparisons. Each phenotype was derived from the *DIS-III-R* depression module according to *DSM-III-R* criteria. Significant results are highlighted in bold. For comparisons which identified a significant result, an additional comparison is presented using the cohort containing no related samples and limiting to controls to individuals with no lifetime history of depression. (B) Results of the Fisher’s Exact Test for single-variant associations based on non-silent rare-variants within genes previously associated with depression. Groups and comparisons are as described above. In both cases the cohort was limited to individuals of European descent. False discovery rate was used to correct for multiple testing in each case using the Benjamini–Hochberg procedure. Full results and allele count for each test type are listed in Supplemental Table 2.

## Data Availability

Investigators interested in accessing phenotypic and genotypic data from the VET Registry (https://www.seattle.eric.research.va.gov/VETR/Home.asp) can apply for data access by contacting the VET Registry at VETR@va.gov.
