# Supplementary material for: Rare variant association study of veteran twin whole-genomes links severe depression with a nonsynonymous change in the neuronal gene BHLHE22
Source: World J Biol Psychiatry. Author manuscript; Available in PMC 2023 Mar 1. (PMC9148382; doi:10.1080/15622975.2021.1980316)
Supplement: Supplement 1 [file NIHMS1773020-supplement-Supplement_1.docx]

Supplementary Materials for

**Rare variant association study of Veteran twin whole-genomes links severe depression with a nonsynonymous change in the neuronal gene BHLHE22**

Daniel Hupalo, Christopher Forsberg, Jack Goldberg, William S. Kremen, Michael J. Lyons, Anthony R. Soltis, Coralie Viollet, Robert J. Ursano, Murray B. Stein, Carol E. Franz, Yan V. Sun, Viola Vaccarino, Nicholas L. Smith, Clifton L. Dalgard, Matthew D. Wilkerson, Harvey B. Pollard

Correspondence to: Harvey Pollard , B2040Uniformed Services University 4301 Jones Bridge Road, Bethesda, Maryland 20814, 301-295-3661, harvey.pollard@usuhs.edu

**This file includes:**

Supplementary Figures S1 to S2


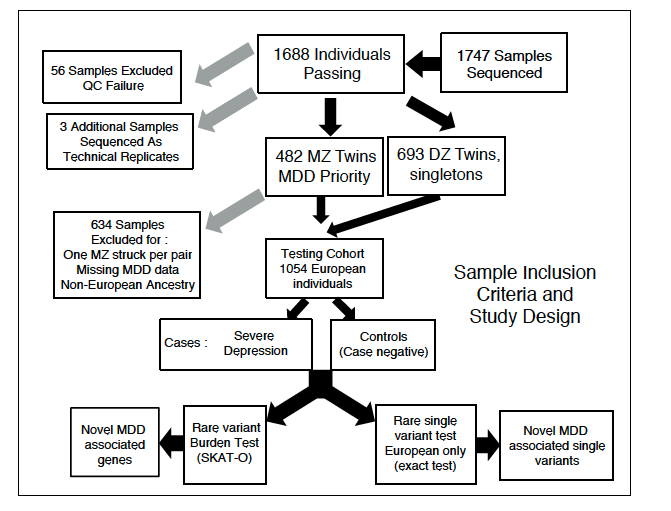


**Supplemental Figure 1.** Experimental design flow chart for calculating risk score and testing for an association with MDD within the VET Cohort.


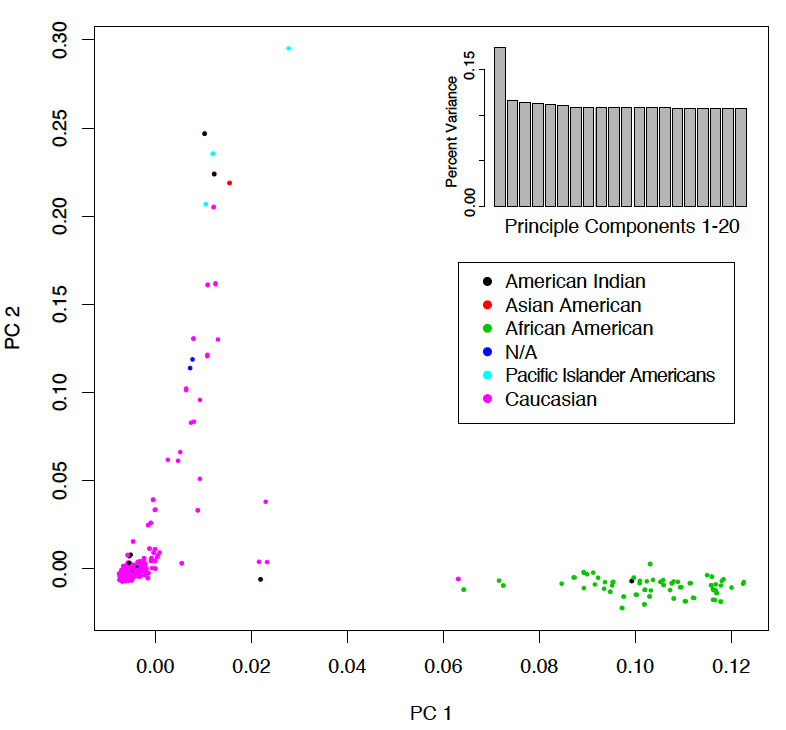


Supplemental Figure 2. Principle component analysis of all individuals within the VET Registry cohort. Variation data from all chromosomes is plotted for PC1 and PC2, with points labeled by self-reported ancestry. Inset are the percent variances for the first 20 principle components.
